# Supplementary material for: Genome-Wide Identification of the CER Gene Family and Significant Features in Climate Adaptation of Castanea mollissima
Source: Int J Mol Sci. 2022 Dec 19;23(24):16202. doi: 10.3390/ijms232416202 (PMC9787725; doi:10.3390/ijms232416202)
Supplement: Supplementary file 1 [file ijms-23-16202-s001.zip › FigureS1 S2 TableS1 S2.pdf]

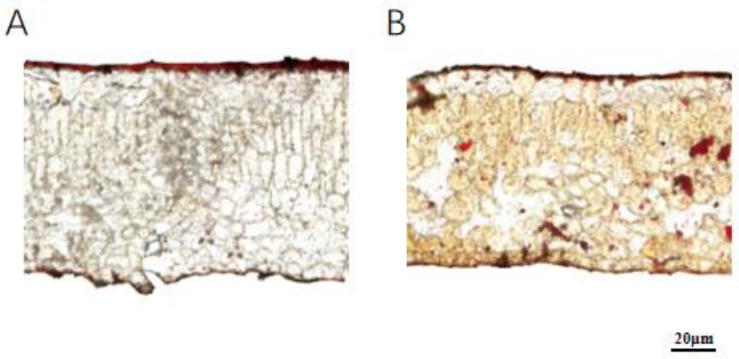

Figure S1 Complete semi-thin section of Gansu and Tianmu Mountain samples. (**A**) Gansu sample. (**B**) Tianmu mountain samples.

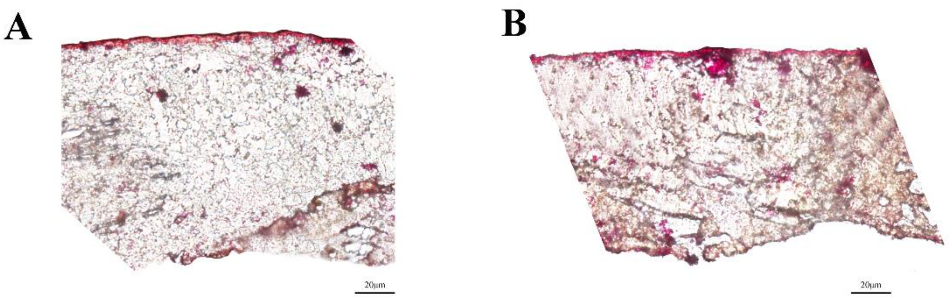

Figure S2 Complete semi-thin section of Y-2_grafted and Y-2_wild samples. (**C**) Y-2_Y-2_grafted sample. (**D**) Y-2_wild sample.

Table S1 Prediction of CmCER family gene cis-elements.

GENE ID	Environmental stress			Plant hormones					MYB binding site		
	Defense and stress	Light	Low temperature	ABA	Aux	Gas	MeJA	SA	Drought inducibility	Flavonoid biosynthesis	MYBHv1 binding site
CmCER 1-1		17		6		2					
CmCER 1-2		11		1	1	1	2		2		
CmCER 1-3		7		3		1	2				
CmCER 1-4	1	14	1	3					1		
CmCER 1-5		13					4	1	1		
CmCER 1-6		10	1	1			2				
CmCER 1-7		16		3			2				
CmCER 1-8	1	13		1	1		2				
CmCER 1-9		6				1	4	1			
CmCER 2	2	15			1	1	2		2		
CmCER 3		12		1					1	1	
CmCER 4		13		3	1		2		1		
CmCER 5-1		18	1	4					1		
CmCER 5-2		7				1					
CmCER 6-1		10		4		2	2		1		1
CmCER 6-2		8	2	2	1		2	1	1		2
CmCER 7		7			4	1			1		1
CmCER 8	1	10		3		1	4	1	1		1
CmCER 9-1		16	2	5		1	8	2			

CmCER 9-2	1	10		2					2		
CmCER 10-1	1	6	1			2	6				
CmCER 10-2		13					2			1	
CmCER 11-1		11		2		1	4				
CmCER 11-2		7	1	1		5	4	1			
CmCER 13	1	9	1	2	1				2		
CmCER 17	1	15		3			4				
CmCER 60-1		13					2				1
CmCER 60-2	1	11		1		2		1	1		
CmCER 60-3	1	10	2	5			4	1	1		
CmCER 60-4	1	8	1	4			4	1	1		
CmCER 60-5		11		1	1	1	2				1
CmCER 60-6		17		2	2	3			2		
CmCER 60-7	2	12	1	1		2	2	1	1		
CmCER 60-8		21	3	2			6	2	2		

Table S2 qPT-PCR primers of CmCER family

GENE ID	Forward Primer 5'-3' sequence	Reverse Primer 5'-3' sequence
CmCER1-1	ACTGTTACTACCATAGCCCTCC	CACTACTCACGAACCGCA
CmCER1-2	ACTGTTACTACCATAGCCCTCC	ACCGCACTCGTGTTCATT
CmCER1-3	TGTTACTACCATAGCCCTCC	TTGCTTGCCAAACTTTCT
CmCER1-4	CATCTTGACGGGAACTGC	ACTTGAGCGGAGGAAACA
CmCER1-5	GGAGGAACTTAACGGATAT	CAGGCAACCTTAGTGATT
CmCER1-6	ACAAGGGCATAATGACTC	GTTACTTCCATCCACCAC
CmCER1-7	TGATTGAGCAAGCCATA	GCTACTTCCATCTACCACC
CmCER1-8	CCGCACCAATTACTCACT	GCAAATCCTAGCCGTAGA
CmCER1-9	GCACCGCCATTATCTTTA	TATGTGAGGTAGCCAGCA
CmCER2	TCCCAATCTTTGAATCTC	ACCCGTCTCCTTTGTTAT
CmCER3	TGAGGAATCCCTAAACGC	CCCAGTCCCATTCTTTGT
CmCER4	GTCTAAAGGTGCTCATCC	ATGTCTAATCCAAGGGTC
CmCER5-1	ACAAGAAGGCCCAGTCAA	TAGCCAGTGCCAACATCA
CmCER5-2	TCAGCCAAGTAGTGAGGT	TATACAACGGAGGAAGTG
CmCER6-1	GATGGGTCCTGATGAAAT	GAAGTAAACAGTGGCAATAA
CmCER6-2	ACATAACGACCAGACCCA	GAAACTCCAACGACGATT
CmCER7	AAGAACAGGGCTCAAGGG	GTTTCTGCCGCCTCAATA
CmCER8	GATGCTTGGATGGCGTAA	CCACTGAGGGCAGTTTGA
CmCER9-1	ATTGGTTCATTGGGTTGT	TCACGGAAAGGATTGTAG
CmCER9-2	TTGGTTCATTGGGCTGTA	TCACGGAAAGGATTGTAG
CmCER10-1	GTCATCCACCCAGTTCAG	AGTAAGCAATGTAAGAGCC
CmCER10-2	TCGGAGCAGTGGAGAAAT	CAGGTTGGACGGGTAGAG
CmCER11-1	ATGAGTCGTTTAGGGTTT	GAGAATAGCGAGTGGAGTA
CmCER11-2	ACTCTGGACGAGGTGTTT	GCTTCCCTTCTTGTTCTA
CmCER13	GGGGCTTCAAGAGGATTA	GGATGATGGCTGGTGATT
CmCER17	CCCACAGGAGTTTCAAGG	AAGCGGTATCAAAGAGCC
CmCER60-1	CCTTGTCTTTCTATCAACCCTT	CCTGCCTTGTGCATTTCC
CmCER60-2	TTCTGTCCGCAATAGTCAA	GTAAGGGATGGTGTAGGG
CmCER60-3	TGGGAGGTGATGAGGAAT	GTTTGAAGGTGGCTGGGT
CmCER60-4	GGAAGGACTGCATTGAAGGGTA	TGGGCGTTTGAAGGTGGC
CmCER60-5	ATAAGTTCCGAATGAGAAG	CTGACAATTAGAGCCAAA
CmCER60-6	CTTGTTGTCACTACCGAGTC	AAAGCATTGCATACCCAC
CmCER60-7	GCAGTGCGGGACTTATTT	TTGCTCGATTCGACAACA
CmCER60-8	GTATGAATTGGCTTACTCTG	GCTGACACTTTAGGCACA
